# Functional importance of Ser323 in cysteine desulfhydrase and cystathionine gamma-lyase MccB of *Staphylococcus aureus*

**DOI:** 10.71150/jm.2411026

**Published:** 2025-02-27

**Authors:** Dukwon Lee, Hyojeong Lee, Kyumi Byun, Eun-Su Park, Nam-Chul Ha

**Affiliations:** 1 Research Institute of Agriculture and Life Sciences, Department of Agricultural Biotechnology, CALS, Seoul National University, Seoul 08826, Republic of Korea; 2 School of Biological Sciences, Institute of Molecular Biology and Genetics, Seoul National University, Seoul 08826, Republic of Korea; 3 The Internship Program at the Food Biochemistry Laboratory, Department of Agricultural Biotechnology, CALS, Seoul National University, Seoul 08826, Republic of Korea; 4 Center for Food and Bioconvergence, Interdisciplinary Programs in Agricultural Genomics, CALS, Seoul National University, Seoul 08826, Republic of Korea

**Keywords:** MccB, cystathionine gamma-lyase (CGL), *Staphylococcus aureus*, pyridoxal 5'-phosphate (PLP), internal aldimine

## Abstract

Pyridoxal 5'-phosphate (PLP)-dependent enzymes participate in various reactions involved in methionine and cysteine metabolism. The representative foodborne pathogen *Staphylococcus aureus* expresses the PLP-dependent enzyme MccB, which exhibits both cystathionine gamma-lyase (CGL) and cysteine desulfhydrase activities. In this study, we investigated the role of Ser323 in MccB, a conserved residue in many PLP-dependent enzymes in the transsulfuration pathway. Our findings reveal that Ser323 forms a hydrogen bond with the catalytic lysine in the absence of PLP, and upon internal aldimine formation, PLP-bound lysine is repositioned away from Ser323. Substituting Ser323 with alanine abolishes the enzymatic activity, similar to mutations at the catalytic lysine site. Spectroscopic analysis suggests that Ser323 is essential for the rapid formation of the internal aldimine with lysine in wild-type MccB. This study highlights the crucial role of Ser323 in catalysis, with broader implications for other PLP-dependent enzymes, and enhances our understanding of the molecular mechanisms involved in the selective control of foodborne pathogenic bacteria.

## Introduction

Pyridoxal 5'-phosphate (PLP)-dependent enzymes play a central role in several metabolic pathways involving amino acids and catalyze various chemical reactions, including transamination, racemization, aldol cleavage, alpha-decarboxylation, beta- and gamma-elimination, and replacement reactions (Du & Ryan, 2019; Hayashi, 1995; Vacca et al., 2008). The enzymes typically feature a conserved lysine residue at the active site, whose ε-amino group makes Schiff base with the aldehyde group, commonly referred to as an internal aldimine (Evande et al., 2004; Schneider et al., 2000)

*Staphylococcus aureus*, a gram-positive bacterium, can cause a variety of infections in humans, ranging from skin irritation to severe diseases such as pneumonia, sepsis, and food poisoning (Le Loir et al., 2003; Lowy, 1998). A key element in the survival and virulence of this bacterium is its metabolic flexibility, which allows it to adapt to diverse hostile environments. MccB is a bifunctional cystathionine gamma-lyase/homocysteine desulfhydrase involved in the conversion of methionine to cysteine via cystathionine gamma-lyase (CGL) activity. MccB also produces hydrogen sulfide from cysteine through its desulfhydrase activity (Binkley and Okeson, 1950; Hullo et al., 2007; Kraus et al., 2009). According to Grishin et al. (1995) MccB are categorized under fold-type I PLP-dependent enzymes, with many enzymes acting via methionine-cysteine metabolic pathways. Intriguingly, recent studies have suggested a link between the cysteine desulfhydrase activity of MccB from *S. aureus* (SaMccB) and antibiotic resistance in *S. aureus*, which is mediated by the production of hydrogen sulfide in the intestine (Luhachack and Nudler, 2014; Shatalin et al., 2021). Notably, hydrogen sulfide can act as a signaling molecule within the gut environment, similar to nitric oxide (NO), a well-known gaseous signal mediator in intestinal physiology (Jeong, 2023; Wang, 2012). Thus, developing inhibitors of MccB could be crucial in controlling bacterial resistance to antibiotics.

The enzymatic function of MccB is fundamentally dependent on the structural and functional characteristics of its active site. Specifically, the interaction between the active site residues and the cofactor PLP is essential for catalysis, as it facilitates the formation of a stable PLP-Schiff base intermediate, which is critical for the enzyme's dual desulfhydrase and cystathionine lyase activities. Our earlier research showed that MccB has a relatively low binding affinity for PLP compared with other PLP-dependent enzymes (Byun et al., 2024). We also discovered that Epigallocatechin gallate (EGCG) specifically inhibited the activity of SaMccB by binding to and removing excess PLP from the solution rather than binding directly to SaMccB (Byun et al., 2024).

The Schiff base of PLP with lysine residues at the active sites of PLP-dependent enzymes is highly stable, whereas the Schiff base with non-catalytic lysine residues is unstable and readily hydrolyzed (Toney, 2011; Tramonti et al., 2022). However, the molecular mechanism underlying specific Schiff base formation at the active sites of the enzymes remains to be elucidated. In this study, we investigated the molecular mechanisms by which the PLP-Schiff base is stabilized in SaMccB, focusing on Ser323 and its equivalent residues. This study aimed to provide a detailed understanding of the molecular mechanisms of PLP-dependent enzymes and their broader roles in biological processes, including hydrogen sulfide-mediated antibiotic resistance.

## Materials and Methods

### Plasmid construction and protein purification

The plasmid for the expression of wild-type SaMccB was used as described in a previous study (Lee et al., 2019). The expression plasmids for the mutant SaMccB proteins (S323A and K196A) were constructed using the Quikchange^®^ site-directed mutagenesis method (Agilent Technologies, USA). The overexpression and purification of the protein were performed according to the procedures previously reported for wild-type SaMccB protein (Byun et al., 2024). In the final size exclusion chromatography step, 20 mM HEPES buffer (pH 7.5) containing 150 mM NaCl was used.

### Crystallization of wild-type and mutant proteins of SaMccB

Wild-type MccB protein (15 mg/ml) in 20 mM HEPES buffer (pH 7.5) containing 150 mM NaCl, 400 μM PLP, and 1 mM cysteine was used to obtain wild-type PLP-bound MccB crystals. The reservoir solution contained 0.1 M sodium acetate trihydrate (pH 4.5) and 30% PEG 300. The S323A mutant SaMccB crystals were obtained from the protein in 20 mM HEPES buffer (pH 7.5) with 150 mM NaCl and 400 μM PLP, using a reservoir solution of 0.1 M Tris buffer (pH 8.5) and 2 M ammonium sulfate. The K196A mutant SaMccB crystals were derived from the protein in 20 mM HEPES buffer (pH 7.5) containing 150 mM NaCl, 400 μM PLP, and 1 mM cysteine, with a reservoir solution of 0.1 M sodium citrate (pH 5.5) and 22% PEG 1000. All crystals were grown at 14°C using the hanging drop vapor diffusion method.

### Data collection and structural determination

X-ray diffraction datasets were collected on beamline 5C at the Pohang Accelerator Laboratory (Korea) at a wavelength of 1.0000 Å (Park et al., 2017). The beamline was equipped with an EIGER-9M detector (DECTRIS, Switzerland). SaMccB crystals were soaked for 5 s in a cryoprotectant buffer containing 25% glycerol in their respective reservoir solutions and then flash-cooled in liquid nitrogen at -196°C. Diffraction datasets were processed using the HKL-2000 program (Otwinowski and Minor, 1997). Molecular replacement was performed to obtain phase information for wild-type PLP-bound SaMccB using the CCP4 program (Winn et al., 2011) with the previously solved SaMccB structure (PDB code: 6KHQ) as the search model (Lee et al., 2019). The S323A mutant MccB structure was solved by molecular replacement using the wild-type apo-type SaMccB structure (PDB code: 6KGZ) determined in this study as the search model. The K196A mutant SaMccB structure was determined using the PLP-bound MccB structure determined in this study as the search model. All the structures were refined using Coot and Phenix (Adams et al., 2010; Emsley and Cowtan, 2004). The statistics for data collection and model refinement are summarized in Table 1.

### Cysteine desulfhydrase activity assay

The cysteine desulfhydrase activity of SaMccB protein was measured using black 96-well plates and hydrogen sulfide-selective fluorescent probe 7-azido-4-methylcoumarin (AzMC), with fluorescence detected using Varioskan Lux Multimode microplate reader (Thermo Fisher Scientific, USA), following a previously described protocol (Byun et al., 2024). Enzymatic assays were conducted with SaMccB (10 μM/well) in 100 mM sodium phosphate buffer (pH 7.0) containing 150 mM NaCl and 100 μM AzMC. Reaction mixtures were plated onto Corning^®^ 96-well black polystyrene clear bottom microplates (CLS3603; Sigma-Aldrich, USA) and incubated with pulsed shaking at 300 rpm. The reaction was initiated by adding cysteine to the final concentration of 100 μM and incubating at 37°C. AzMC fluorescence was monitored at excitation and emission wavelengths of 365 and 450 nm, respectively, with readings taken every 20 s. All data were analyzed and visualized using GraphPad Prism 8 (GraphPad Software Inc., USA).

### CGL activity assay

Canonical CGL activity utilizing cystathionine was measured using purified SaMccB, following a previously described protocol (Byun et al., 2024). The enzymatic activity was assessed using the substrate cystathionine by monitoring the production of cysteine. The amount of cysteine generated was quantified using 5,5′-dithiobis-(2-nitrobenzoic acid) (DTNB), which reacts with cysteine to produce the chromogenic compound 2-nitro-5-thiobenzoate (TNB). CGL activity assays using SaMccB (10 μM) were conducted in 100 mM sodium phosphate buffer (pH 7.0) containing 150 mM NaCl and 100 μM DTNB. The reaction was initiated by adding cystathionine to the final concentration of 100 μM. The absorbance of TNB was recorded at 415 nm at 20-s intervals using Varioskan Lux Multimode microplate reader (Thermo Fisher Scientific, USA), and the enzymatic activity was determined from the slope during the linear phase of increased fluorescence intensity. All data were analyzed and visualized using GraphPad Prism 8 (GraphPad Software Inc., USA).

### UV-Vis absorption spectroscopy

The protein sample (50 μM) in 100 mM sodium phosphate (pH 7.0) buffer containing 150 mM NaCl was transferred to a clean quartz cuvette with a path length of 1 cm. Subsequently, 50 μM PLP was added to the cuvette. The UV-Vis absorption spectra were recorded using a pre-calibrated UV-Vis spectrophotometer (GE GeneQuant 1300 UV/Vis Spectrophotometer). Measurements were performed over a wavelength range of 250–500 nm every 10 s for 180 s at room temperature (25°C). The spectra were analyzed using Microsoft Excel, and the absorbance was recorded at 420 nm, which represents the Schiff base of PLP (Morino and Nagashima, 1984).

### Phylogenetic tree

The amino acid sequences of PLP-dependent enzymes from various organisms were retrieved from the Universal Protein Resource (https://www.uniprot.org) using BLAST, with the query sequence being that of SaMccB from the PDB-deposited data. Multiple sequence alignment was performed using Clustal Omega, integrated into Mega X (Kumar et al., 2018). The evolutionary history was inferred using the Maximum Likelihood (ML) method, with the phylogenetic tree constructed based on 1,000 bootstrap tests in Molecular Evolutionary Genetics Analysis (Mega) version 11 (Kumar et al., 2018). The analysis included 35 amino acid sequences.

## Results

### Wile-type SaMccB structure in PLP-covalently linked form

**Fig. 1.**
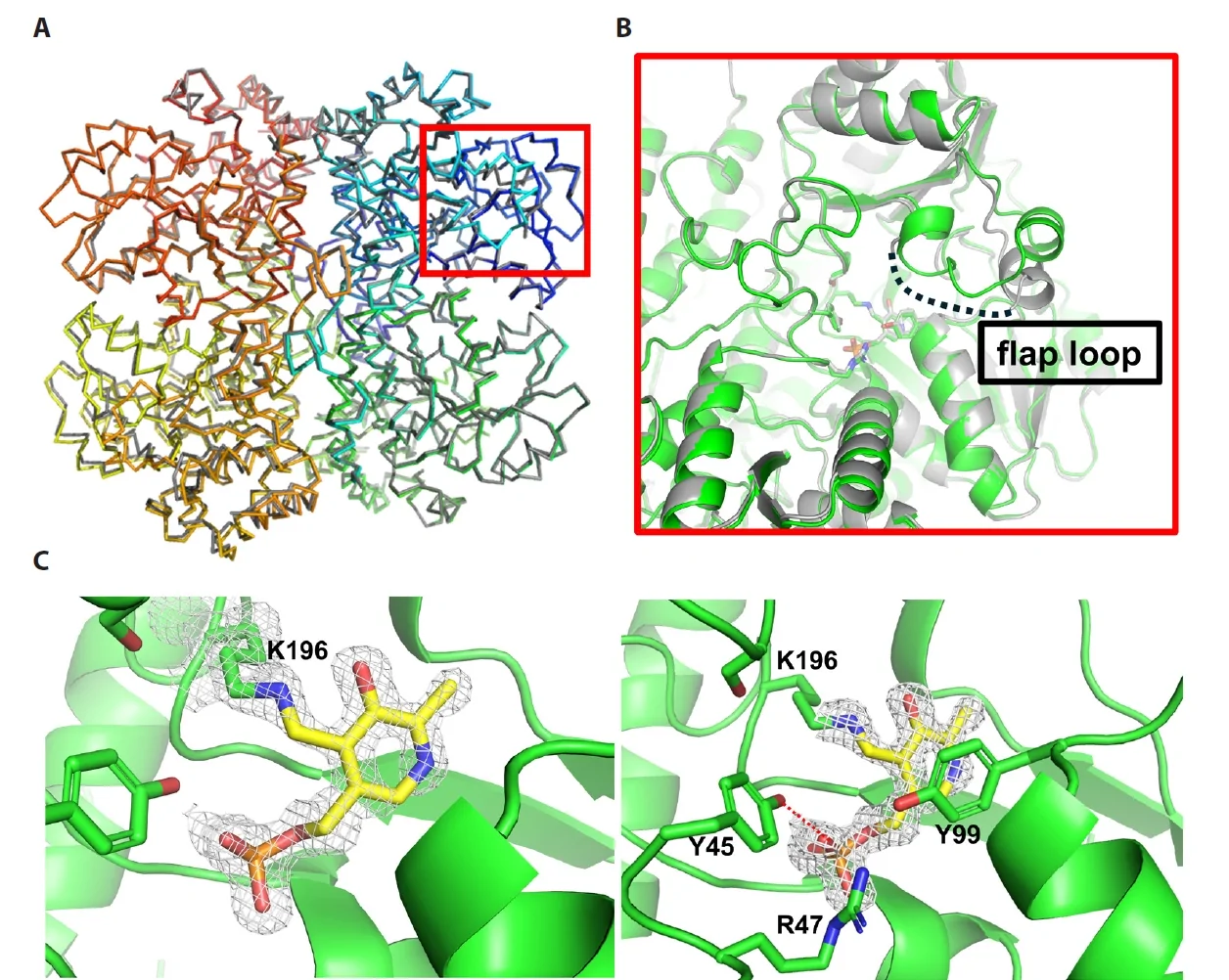
Crystal structure of PLP-bound SaMccB in the internal aldimine form. (A) Structural superposition of PLP-bound SaMccB at 1.64 Å resolution (this study) with the previously reported structure (PDB code: 6KHQ, gray [Lee et al., 2019]) in the Cα representation. In this study, PLP-bound SaMccB was distinct from its protomers in the rainbow coloring. Red rectangle indicates the active site of MccB. (B) An enlarged view of the active site region indicated by the red rectangle in Fig. 1A. The disordered flap loop region in the previous low-resolution structure is indicated by a broken line. (C) Electron density map of PLP-bound Lys196 in the internal aldimine state. The map is contoured at 1.0 sigma using the 2FoFc dataset. The left panel highlights the PLP molecule, while the right panel illustrates the chemical environment of the bound PLP. Key residues, including Tyr45, Arg47, and Tyr99, are depicted as stick models.

SaMccB exists in three distinct states: PLP-free, PLP-internal aldimine, and PLP-external aldimine forms. The crystal structures of SaMccB in both PLP-free and PLP-covalently linked states at 2.3 Å resolution are available (Lee et al., 2019). To better define the PLP-covalently linked or internal aldimine structure, we updated the crystal structure of SaMccB in this state at a higher resolution. We determined the structure of SaMccB in the presence of PLP in reaction buffer, revealing the internal aldimine form at a resolution of 1.64 Å. The higher resolution structure exhibited a nearly identical conformation to the previous one (rmsd = 0.253 Å) and even showed the flap loops (residues 338–342) around the active site, which was disordered in the structure at a resolution of 2.3 Å (Fig. 1A & 1B). However, the potential reaction of bound PLP at the active site appeared to result in a disrupted electron density map for PLP (Fig. 1C).

We compared a previously reported PLP-free structure (Lee et al., 2019) with PLP-bound internal aldimine structure described in this study. In the PLP-free form, Ser323 forms a hydrogen bond with Lys196, with a 3.0 Å distance between the side chain hydroxyl group of Ser323 and the side chain amine group of Lys196. Ser323 also participates in the hydrogen bond network with the neighboring water molecules, Ser202, and Glu328 residues (Lee et al., 2019).

**Fig. 2.**
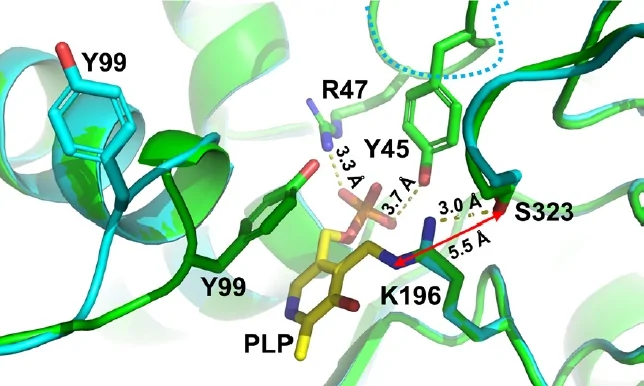
Structural comparison of PLP-bound SaMccB with apo-SaMccB at the active site. The structure of apo-SaMccB (PDB code: 6KGZ [Lee, et al., 2019]) is shown in cyan, whereas PLP-bound structure from this study is shown in green. Hydrogen bonds involving the phosphate group of PLP (yellow sticks) are depicted as dashed lines, with the bond lengths indicated. The distance between the aldimine nitrogen and the hydroxyl group of Ser323 is marked by a red arrow, indicating a distance of 5.5 Å. The loop containing Tyr45 and Arg47 in apo-MccB is disordered and represented by a cyan dashed line.

Upon PLP binding, the pyridoxal ring is stabilized by the aromatic ring of Tyr99, accompanied by a conformational change in the active site loop (residues 37–48) (Fig. 1C), a feature observed in many PLP-dependent enzymes (Huai et al., 2001). Specifically, Tyr45 and Arg47 in the active site loop interact with the phosphate group of PLP. Notably, the distance between the side chain hydroxyl group of Ser323 and the internal aldimine group of Lys196 increases to 5.5 Å, making PLP molecule inaccessible (Fig. 2).

### Ser323 plays a crucial role in enzymatic activities

**Fig. 3.**
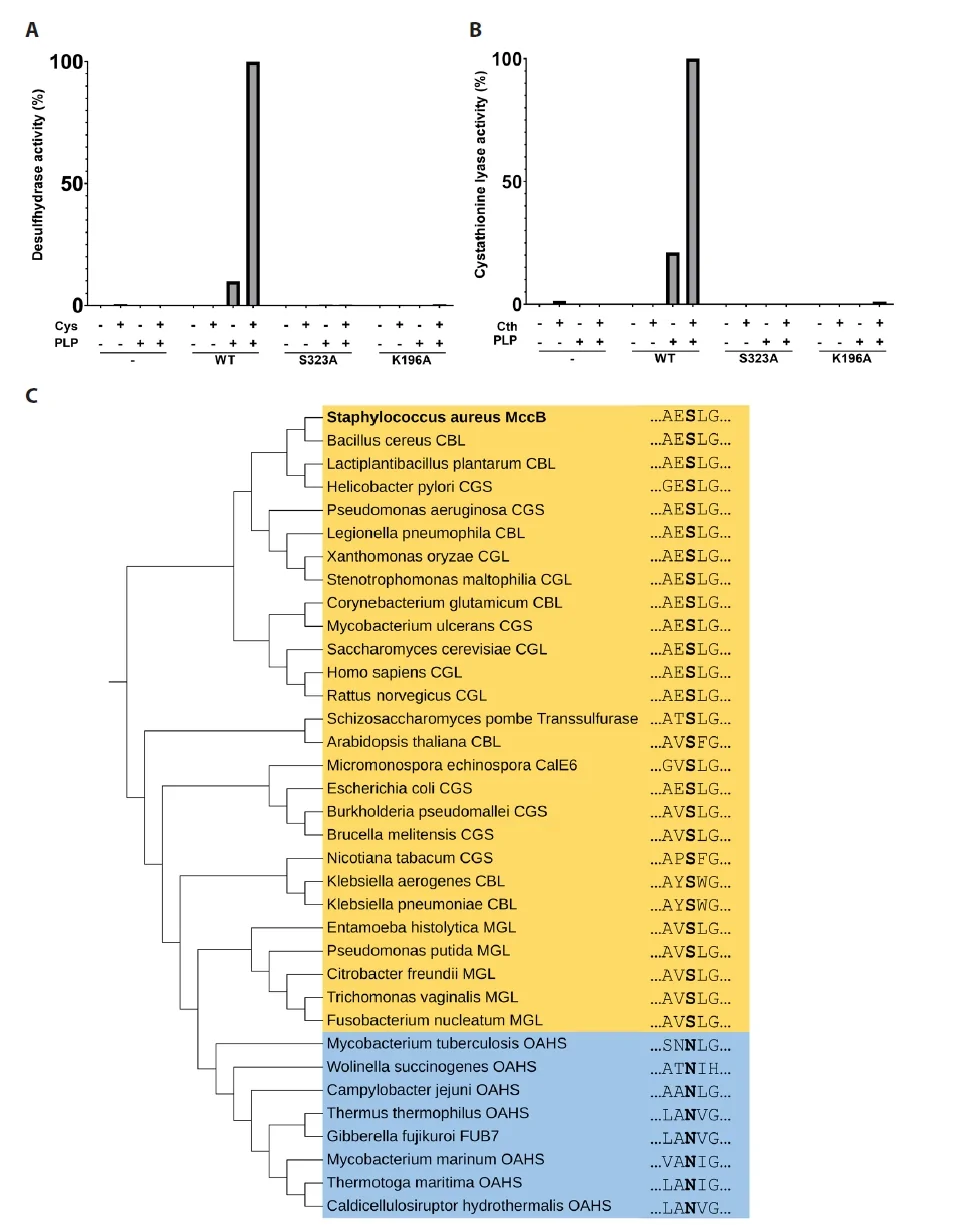
Importance of Ser323 in enzymatic activities of SaMccB. (A) Cysteine desulfhydrase activity of wild-type and S323A and K196A mutants of SaMccB. The activities were measured using cysteine (Cys) as the substrate in the presence of PLP in buffer. The reactions were monitored using the hydrogen sulfide-reacting agent, AzMC, and fluorescence was measured at an excitation wavelength of 365 nm and an emission wavelength of 450 nm. The results are presented as relative ratio to the initial reaction rate. (B) CGL activity of wild-type and S323A and K196A mutants of SaMccB. Enzymatic reactions were performed using cystathionine (Cth) as the substrate in the presence of PLP in buffer. The amount of cysteine produced was quantified by measuring the absorbance at 412 nm using the DTNB assay. The results are presented as relative ratio to the initial reaction rate. (C) Phylogenetic tree analysis with a focus on Ser323. Fold-type I PLP-dependent enzymes were analyzed, and a phylogenetic tree was constructed using Mega X (Kumar et al., 2018). The five amino acids surrounding Ser323 and their equivalent residues are displayed along with their respective protein names. The S-group containing a serine residue at this position is highlighted in yellow, whereas the N-group containing an asparagine residue is shaded in cyan. CGL, cystathionine gamma-lyase; CBL, cystathionine beta-lyase; CGS, cystathionine gamma-synthase; MGL, methionine gamma-lyase; OAHS, O-acetyl-homoserine sulfhydrylase.

To investigate the role of Ser323, we generated mutant enzymes and evaluated their activity. Cysteine was incubated with SaMccB as the substrate in the presence of the cofactor PLP at 25°C, and the reaction product, hydrogen sulfide, was quantified using the hydrogen sulfide-reacting agent AzMC. The mutation of Ser323 to alanine (S323A) dramatically decreased the enzymatic activity of MccB at pH 7.0 (Fig. 3A). We performed the same experiment using the K196A mutant MccB protein to confirm the role of Lys196. The Lys196 mutation completely abolished the enzymatic activity (Fig. 3A). These results highlighted the critical importance of Ser323 in the enzymatic activity of SaMccB.

Next, we examined the effect of S323A mutation on the CGL activity of SaMccB. Cystathionine was used as the substrate, and the resulting cysteine was measured to assess the catalytic activity using DTNB. S323A mutation, like K196A mutation, abolished the CGL activity of MccB. These findings highlight the essential role of Ser323 in cysteine desulfhydrase and CGL activities (Fig. 3B).

### Phylogenetic analysis of PLP-dependent enzymes with a focus on Ser323 residue

We analyzed PLP-dependent enzymes with a focus on Ser323 residue using multiple sequence alignment and constructed a phylogenetic tree (Fig. 3C). Among all enzymes, only serine or asparagine residues were found at the Ser323 position. Based on this observation, we classified the enzymes into two groups: The S group, which contained serine, and the N group, which contained asparagine. The N group predominantly includes enzymes such as O-acetyl-L-homoserine sulfhydrylase (OAHS, MetY) and plays a crucial role in the early stages of methionine biosynthesis by regulating the flux of sulfur amino acid synthesis. In contrast, the S group comprises enzymes, such as cystathionine beta-lyase (CBS), cystathionine gamma-lyase (CGL, CSE), and methionine gamma-lyase (MGL, CalE6), which are widely distributed across various organisms and involved in cysteine and methionine metabolic pathways.

### Crystal structures of S323A and K196A mutant SaMccB proteins

**Fig. 4.**
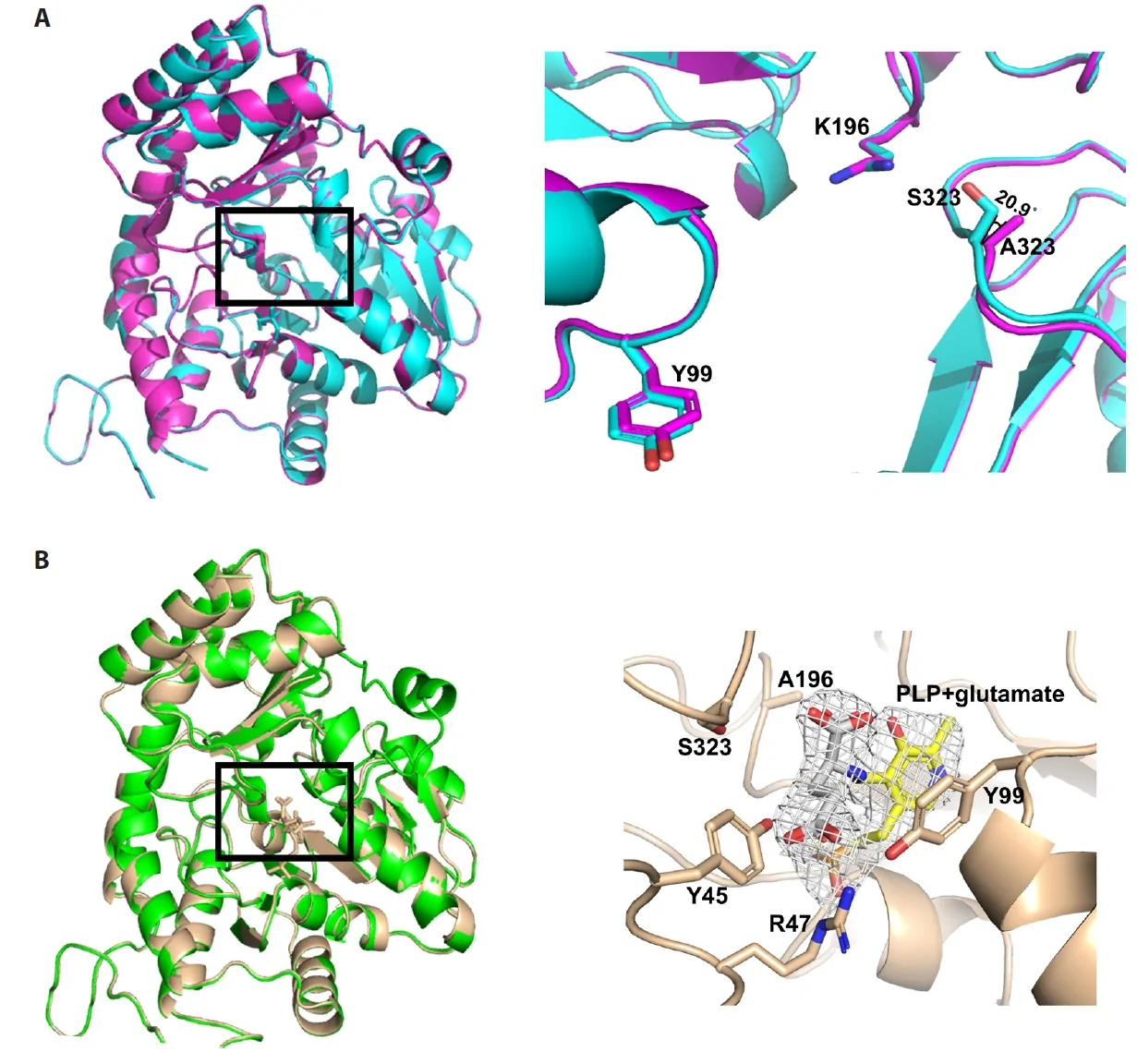
Structural characterization of S323A and K196A mutants of SaMccBs. (A) Structural comparison of apo forms of wild-type and S323A mutant. One probe from each protein sample was superimposed. Wild-type structure is shown in cyan, and S323A mutant is shown in magenta. For clarity, the boxed region on the left is enlarged in the right panel. Tyr99, Lys196, and Ser323 (or Ala323 in the mutant) are shown as sticks. The angular difference between the Cβ atoms relative to the Cα atoms is 20.9°. (B) Structural superposition of the protomers of wild-type (green) and K196A mutant (wheat). Wild-type is in the internal aldimine form, whereas K196A mutant is in the external aldimine form. The boxed regions are shown in the right panel. The electron density map of the putative PLP-aldimine with glutamate is shown in the right-hand panel.

To determine the effect of S323A mutation resulting from structural alterations, we depicted the crystal structure of S323A at a resolution of 2.2 Å using molecular replacement with wild-type apo-type SaMccB structure as the search model. Although PLP was present during crystallization, it was not present in the final crystal structure. The structure of S323A variant was nearly identical to that of wild-type enzyme in the apo-form (Fig. 4A; rmsd = 0.150 Å). Ser323 mutation did not induce significant conformational changes in the active site loops. Notably, the direction of C-beta atom at Ser323 of wild-type protein differed by 20.9° from that of Ala323 in S323A mutant (Fig. 4A). These findings suggest that the structural strain was caused by the oxygen atom of Ser323.

We found that the purified K196A variant of SaMccB protein, expressed in *E. coli*, exhibited an absorption peak at 420 nm in PLP-free buffer (Fig. S1). The crystal structure of K196A protein was subsequently elucidated at 2.5 Å resolution using the molecular replacement method. The overall structure was very similar to that of PLP-bound wild-type SaMccB (Fig. 4B; rmsd = 0.229 Å). Interestingly, the crystal structure revealed the presence of PLP molecule at the active site. Further examination suggested that bound PLP was in its external aldimine form, similar to glutamate (Fig. 4B). We speculated that the external aldimine PLP molecules originated from the culture medium or *E. coli* cytosol. In wild-type enzyme, this external aldimine is likely broken down by the catalytic lysine residue at the active site.

### Ser323 contributes to faster Schiff base complex with PLP

**Fig. 5.**
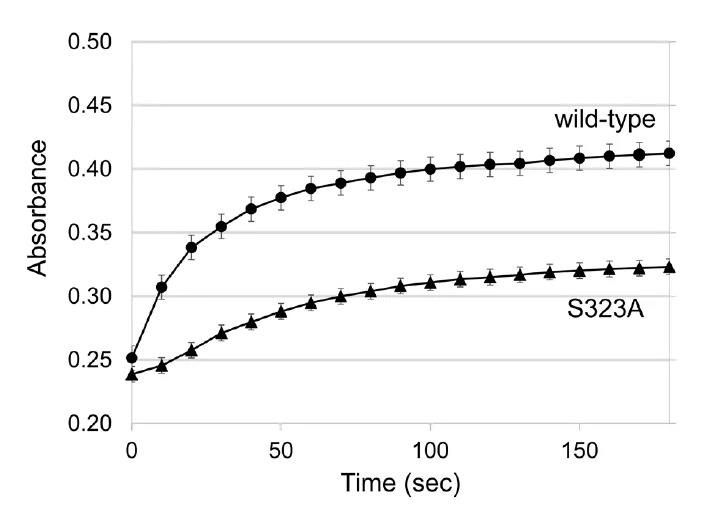
Rates of PLP-Schiff base formation in wild-type and S323A mutant MccB. Absorbance at 420 nm following PLP treatment of wild-type (circle) and S323A mutant MccB (triangle) was measured over 180 s, with data points collected every 10 s. Error bars represent the standard error of three independent experiments. The increase in absorbance at 420 nm indicated Schiff base formation between the PLP and protein samples.

We monitored the binding of PLP using its UV-Vis absorption spectra. PLP in solution exhibited an absorption maximum at 390 nm, whereas the internal aldimine PLP exhibited a maximum absorbance at 415–420 nm (Han et al., 2015; Morino and Nagashima, 1984). We added either wild-type or S323A mutant MccB enzyme to 20 mM HEPES buffer at pH 7.0. UV-Vis spectra were recorded every 10 s at 250–500 nm to measure the absorption maximum (Fig. 5 & S2). Wild-type enzyme showed a 25% higher absorption intensity at approximately 420 nm than that of mutant enzyme. Thus, Ser323 promotes Schiff base formation with lysine residue at the active site of MccB.

## Discussion

While AI-based prediction programs, such as AlphaFold 2, have significantly advanced the field of structural biology, experimental determination of protein complex structures and mutant protein structures remains indispensable (Song, 2023). These experimentally derived structures provide critical insights into the molecular mechanisms of enzymes that cannot be fully captured through computational predictions alone. In this study, we determined the crystal structures of wild-type SaMccB and its S323A mutant. Structural comparisons between the PLP-free form and the PLP-bound internal aldimine complex revealed changes in the distance between the primary amine of the catalytic lysine residue and the hydroxyl group of Ser323. In the PLP-free state, this distance was close enough to form a hydrogen bond; however, it increased in the PLP-bound state owing to structural changes upon PLP binding. Ser323 mutation abolished the enzymatic activity of SaMccB, similar to the effect of mutating the catalytic lysine residue, underscoring the essential role of Ser323 in catalysis. UV-Vis spectroscopy results suggest that Ser323 facilitates the formation of the internal aldimine with PLP.

K196A mutant MccB did not exhibit any enzymatic activity. However, the external aldimine form of PLP was observed in the purified protein using UV-Vis spectroscopy. Furthermore, the external aldimine form of PLP was observed in the active site of K196A mutant structure even though PLP was not added during the crystallization. These observations support the crucial role of the catalytic lysine residue in either hydrolysis or transfer of the amine group involved in the external aldimine formation of PLP.

**Fig. 6.**
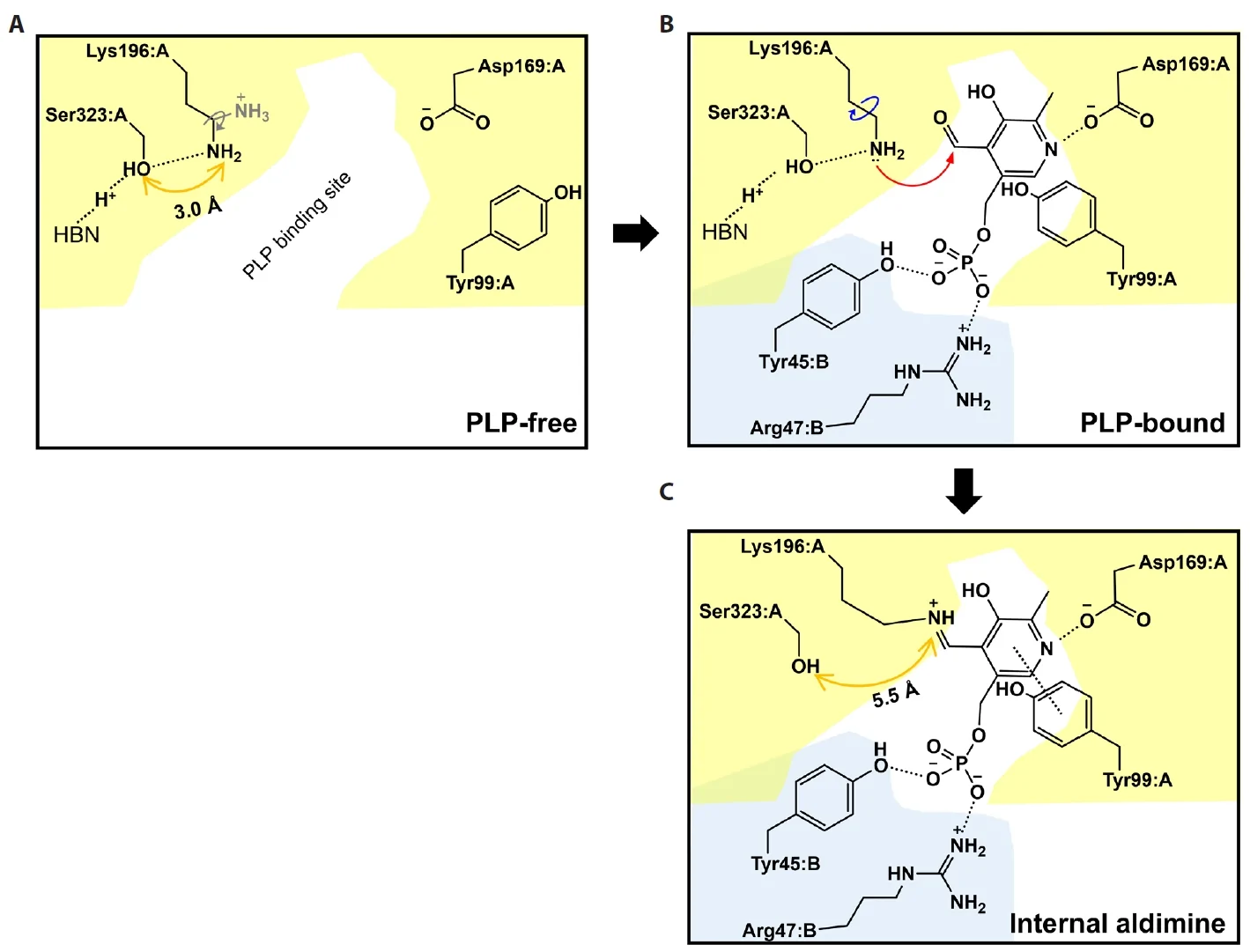
A proposed mechanism of the internal aldimine formation in PLP-dependent enzymes. (A) PLP-free state. In the absence of PLP, Ser323 forms part of a hydrogen bond network (HBN) at the active site. Lys196 is deprotonated by donating a proton to Ser323, which is stabilized in HBN. The yellow shadow indicates the structure of protomer A and the PLP binding site. (B) PLP-bound state. When PLP binds to the active site, Tyr99 flips toward the pyridoxal ring of PLP and is further stabilized by Asp169 at the N^+^ of the pyridoxal ring. The phosphate group of PLP forms polar and ionic interactions with Tyr45 and Arg47 in protomer B (cyan shadow). The amine group of Lys196 performs a nucleophilic attack on the aldehyde group of PLP (red arrow), which is facilitated by the rotational motion of the Lys196 side chain (blue arrow). (C) Internal aldimine state. PLP forms a Schiff base with Lys196, which is known as an internal aldimine. However, this reversible reaction was not facilitated by Ser323 due to its distance from the aldimine nitrogen atom.

Based on these observations, we proposed a mechanism for the formation of a stable internal aldimine at the active site. Both the formation and breakdown of Schiff bases can be accelerated by the presence of a properly aligned general base and acid. In this mechanism, Ser323 acts as a general base, facilitating deprotonation of the catalytic lysine, which is crucial for the formation of Schiff base with PLP (Fig. 6). The crystal structure of MccB reveals that Ser323 is involved in hydrogen bond networking, presumably stabilizing a proton through interactions with Glu328 or water molecules (Lee et al., 2019). However, the study also found that once the internal aldimine is formed, Ser323 is not positioned to influence the protonation or deprotonation of Schiff base owing to the orientation of PLP molecule. Therefore, Ser323 may not facilitate the breakdown of internal aldimine. These findings suggest a structure-based mechanism for stable Schiff base formation in MccB and other Ser323-containing PLP-dependent enzymes.

According to the phylogenetic tree analysis focusing on residues at the Ser323 position, fold-type I PLP-dependent enzymes can be classified into two groups: The S-group and the N-group. Notably, the N-group exclusively includes OAHS, which is involved in the direct sulfurylation pathway for methionine biosynthesis predominantly found in certain bacteria and archaea (Gophna et al., 2005). In this pathway, the precursor molecule O-acetyl-homoserine is directly converted to methionine by the enzyme OAHS, bypassing cystathionine as an intermediate. In contrast, the S-group enzymes are predominantly found in plants, animals, and most bacteria. This group includes enzymes, such as CGL and CBL, which participate in the transsulfuration pathway using cystathionine as an intermediate. All PLP-dependent enzymes share the internal aldimine PLP as the first essential step in their respective reactions. Asparagine has a longer side chain than serine, and differential reactions between the N- and S-groups may be influenced by this difference. Further studies are required to elucidate the molecular roles of Ser and Asn residues in PLP-dependent mechanisms.

In conclusion, this study elucidates the essential role of Ser323 in the catalytic mechanism of PLP-dependent enzymes, particularly SaMccB. Mutation of Ser323 leads to loss of enzymatic activity, mirroring the effects of mutations at the catalytic lysine site. Analysis of the crystal structures of both wild-type and mutant SaMccB proteins suggests a structure-based mechanism for Schiff base formation, emphasizing the significance of Ser323 in PLP-dependent catalysis. These findings advance our understanding of the molecular mechanisms underlying the selective control of foodborne pathogenic bacteria.

## Figures and Tables

**Table 1.** Data collection and refinement statistics of SaMccB structures

Data collection	Wild-type, internal aldimine	S323A variant, apo-form	K196A variant, external aldimine
Beamline	PAL 5C	PAL 5C	PAL 5C
Wavelength (Å)	1.00000	1.00000	1.00000
Space group	*I*222	*I*4_1_22_1_	*C*222_1_
*a*, *b*, *c *(Å)	62.7, 80.3, 161.4	105.2, 105.2, 289.4	56.0, 154.5, 150.0
α, β, γ (°)	90, 90, 90	90, 90, 90	90, 90, 90
Resolution (Å)	50.0–1.64 (1.67–1.64)	50.0–2.19 (2.24–2.19)	50.0–2.5 (2.54–2.50)
*R*_pim_	0.016 (0.086)	0.018 (0.087)	0.022 (0.086)
*I*/σ	6.81	2.54	5.85
Completeness (%)	97.2 (96.4)	99.4 (99.7)	92.0 (84.0)
Redundancy	13.3 (13.3)	15.9 (9.3)	6.4 (3.5)
Resolution (Å)	31.26–1.64	33.26–2.19	36.26–2.5
No. of reflections	48,591	40,789	20,756
*R*_work_/*R*_free_	0.1735/0.2006	0.1932/0.2229	0.2163/0.2660
No. of total atoms	3,165	2,813	5,700
Wilson B-factor (Å)	12.8	25.5	31.5
Bond lengths (Å)	0.002	0.006	0.002
Bond angles (°)	0.511	0.818	0.509
Favored (%)	98.40	97.5	96.5
Allowed (%)	1.33	2.5	3.5
Outliers (%)	0.27	0.0	0.0
PDB code	9J7Q	9J7P	9J7R

## Supplementary Information

Fig. S1. UV-Vis spectroscopy (260–460 nm) results of purified SaMccB wild-type enzyme and S323A, K196A proteins. Green represents wild-type, magenta represents S323A, and wheat represents K196A. The enlarged region between 380 and 450 nm, indicated by the box on the right, shows the aldimine binding signal of PLP. S323A shows no PLP signal, whereas wild- type and K196A display aldimine binding signals, indicating PLP without its addition during the purification process.

Fig. S2. UV-Vis absorption spectra of MccB protein with PLP in 100 mM sodium phosphate buffer containing 150 mM NaCl. "PLP" represents the spectrum of 50 µM PLP without MccB protein, whereas "0–180" indicates mixtures of 50 µM PLP and 50 µM MccB incubated for the specified times (in seconds).
