# Supplementary material for: Functional importance of Ser323 in cysteine desulfhydrase and cystathionine gamma-lyase MccB of Staphylococcus aureus
Source: J Microbiol. 2025 Feb 27;63(2):e2411026. doi: 10.71150/jm.2411026 (PMC13577012; doi:10.71150/jm.2411026)
Supplement: Supplementary file 1 [file jm-2411026-Supplementary-Fig-S1.pdf]

SUPPLEMENTARY FIGURE

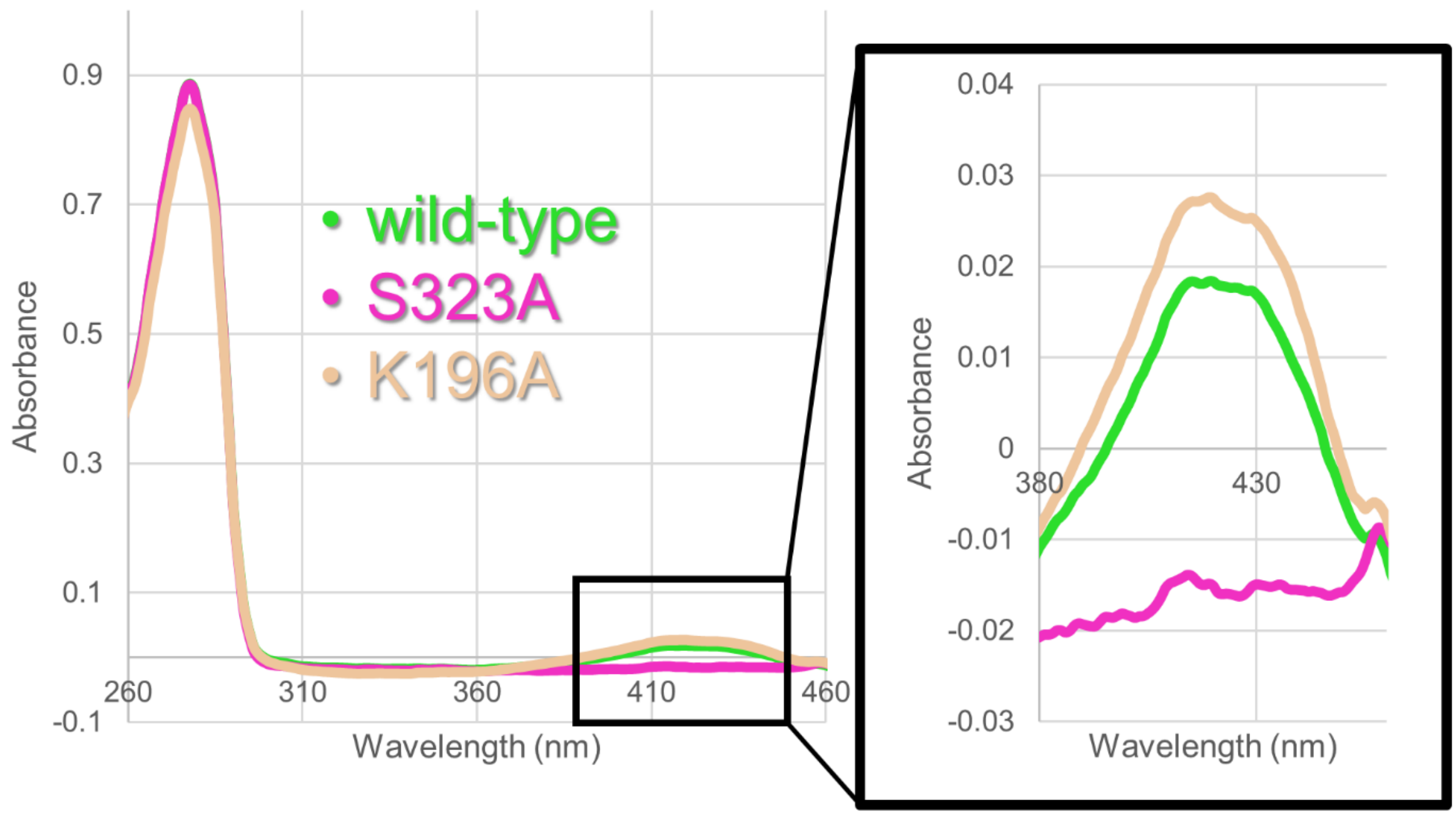

Fig. S1. UV-Vis spectroscopy (260–460 nm) results of purified SaMccB wild-type enzyme and S323A, K196A proteins. Green represents wild-type, magenta represents S323A, and wheat represents K196A. The enlarged region between 380 and 450 nm, indicated by the box on the right, shows the aldimine binding signal of PLP. S323A shows no PLP signal, whereas wild-type and K196A display aldimine binding signals, indicating PLP without its addition during the purification process.
