# Supplementary material for: Functional importance of Ser323 in cysteine desulfhydrase and cystathionine gamma-lyase MccB of Staphylococcus aureus
Source: J Microbiol. 2025 Feb 27;63(2):e2411026. doi: 10.71150/jm.2411026 (PMC13577012; doi:10.71150/jm.2411026)
Supplement: Supplementary file 2 [file jm-2411026-Supplementary-Fig-S2.pdf]

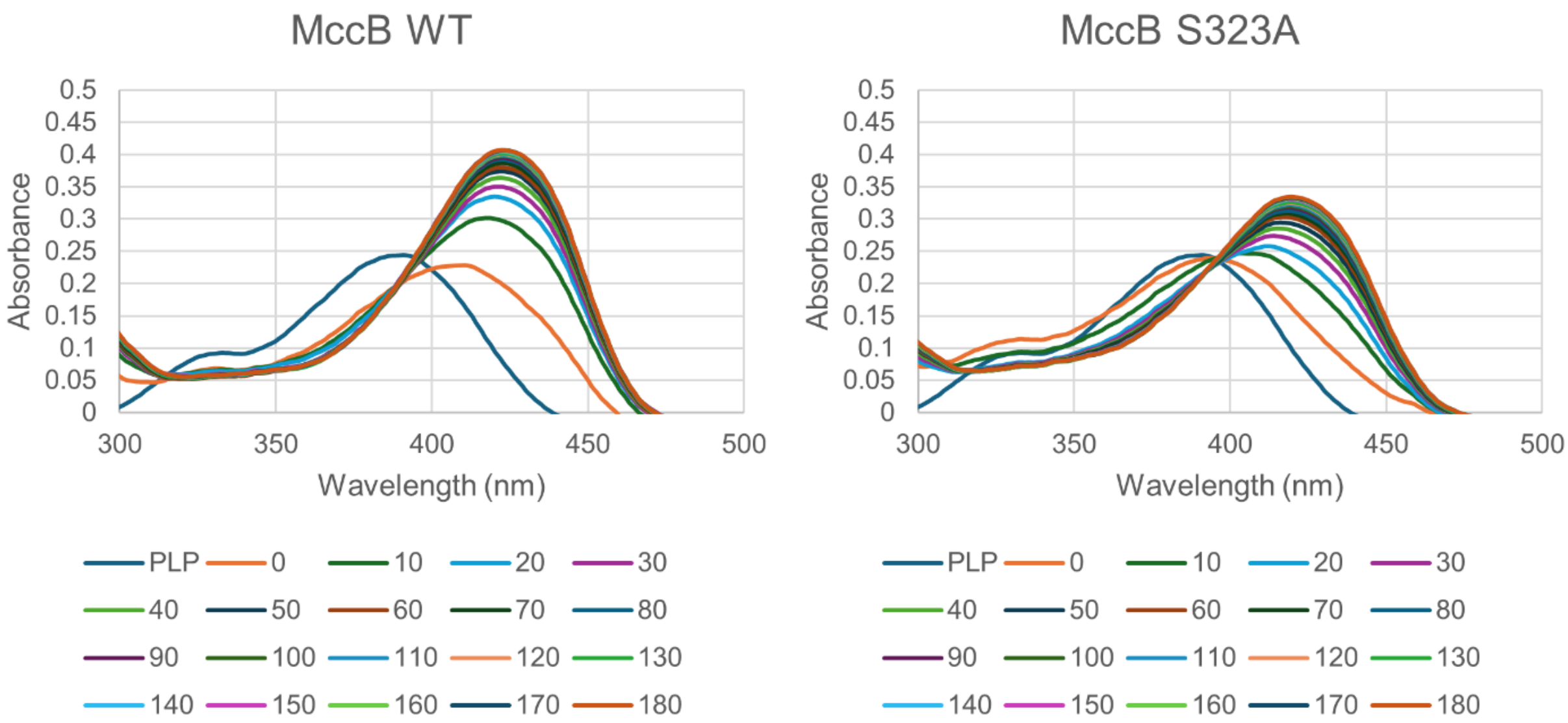

Fig. S2. UV-Vis absorption spectra of MccB protein with PLP in 100 mM sodium phosphate buffer containing 150 mM NaCl. "PLP" represents the spectrum of 50 µM PLP without MccB protein, whereas "0–180" indicates mixtures of 50 µM PLP and 50 µM MccB incubated for the specified times (in seconds).
